# Case Report: A rare case of bilateral primary Müllerian adenosarcoma of the ovary with Uncommon extensive metastasis and *GNAQ* mutation

**DOI:** 10.3389/fonc.2025.1657939

**Published:** 2025-12-03

**Authors:** Shuying Ai, Simin Li, Huihua He, Jingping Yuan, Honglin Yan, Xiuyun Zhang

**Affiliations:** Department of Pathology, Renmin Hospital of Wuhan University, Wuhan, China

**Keywords:** ovary, adenosarcoma, metastasis, lymphovascular invasion, mutation

## Abstract

**Background:**

Müllerian adenosarcoma (MA) is a rare low-grade malignant tumor. It is characterized by benign glandular epithelium and malignant sarcomatous stromal components. This tumor commonly arises in the uterine corpus. Primary ovarian MA is uncommon and typically presents as a unilateral lesion (FIGO stages I-III). However, bilateral ovarian involvement coupled with extensive metastasis in ovarian adenosarcoma has not been previously reported.

**Case presentation:**

This article reports a rare case of bilateral primary ovarian adenosarcoma with extensive metastasis and lymphovascular invasion (LVI). Histopathological examination revealed a biphasic tumor composed of proliferative glands and stroma. The glandular epithelium was separated by abundant stromal components, forming a lobulated architecture resembling a breast phyllodes tumor. The glandular epithelial cells exhibited a single-layered columnar morphology with bland cytological features. In contrast, the stromal cells displayed a spindle-shaped morphology, arranged densely around spiral artery-like vessels. These stromal cells showed mild atypia with visible mitotic figures. Notably, periglandular stromal condensation formed a characteristic “cuff-like” pattern. Extensive metastases were identified in multiple sites: the subumbilical region, intestinal tract, omentum, and distal segment of the right ureter. Significantly, tumor emboli were detected within vascular channels beneath the rectal serosa. Molecular analysis revealed novel findings. Next-generation sequencing (NGS) identified a GNAQ mutation, previously unreported in this tumor type. Additionally, immunohistochemical (IHC) analysis confirmed positive PD-L1 expression. Finally, the patient received a comprehensive treatment regimen comprising radiotherapy, chemotherapy, and immunotherapy.

**Conclusion:**

We describe a unique case of ovarian adenosarcoma presenting with bilateral ovarian involvement, manifesting at the more advanced stage IIIC, notably in the absence of sarcomatous overgrowth (SO). Moreover, we report for the first time the presence of LVI and the *GNAQ* missense variant in ovarian adenosarcoma.

## 1 Background

Müllerian adenosarcoma (MA), a distinctive uterine tumor, was first reported by Clement et al. (1) in 1974. Histologically and biologically, it represents an intermediate entity between benign adenofibromas and highly malignant carcinosarcomas. While uterine adenosarcoma generally portended a favorable prognosis, cases complicated by sarcomatous overgrowth (SO) demonstrated malignant potential (2, 3). SO was defined as pure sarcoma comprising at least 25% of the tumor, typically of high grade and lacking a benign glandular component (4). Although rare, these neoplasms have also been reported in extrauterine sites such as the ovary, vagina, or other locations (5). Ovarian primary cases account for merely 0.04% of ovarian neoplasms (6). Prior studies indicated that ovarian adenosarcoma predominantly manifested as unilateral disease (FIGO stages I-III), with stage distribution analysis revealing 65% at stage I, 27.5% at stage II, and 7.5% at stage III. In addition, SO was significantly associated with disease recurrence or extraovarian dissemination (7). Given the relative rarity of adenosarcomas, most molecular studies have incorporated samples from uterine and extrauterine primary sites, primarily focusing on uterine tumors. Emerging evidence highlighted genetic alterations in the *PI3K/AKT/PTEN* pathway, as well as amplifications of the *MDM2/CDK4* locus (8). SO was more frequently observed in high-grade tumors, which were mainly related to *TP53* mutations and *BAP1* homozygous deletions (9, 10).

Here, we described a unique case of ovarian adenosarcoma with distinctive clinicopathological and molecular features. The case involved bilateral ovarian involvement, presenting at an advanced stage IIIC, yet notably without SO. What’s more, we reported for the first time the presence of lymphovascular invasion (LVI) and a *GNAQ* missense variant in ovarian adenosarcoma.

## 2 Case presentation

### 2.1 Clinical features

**Figure 1:**
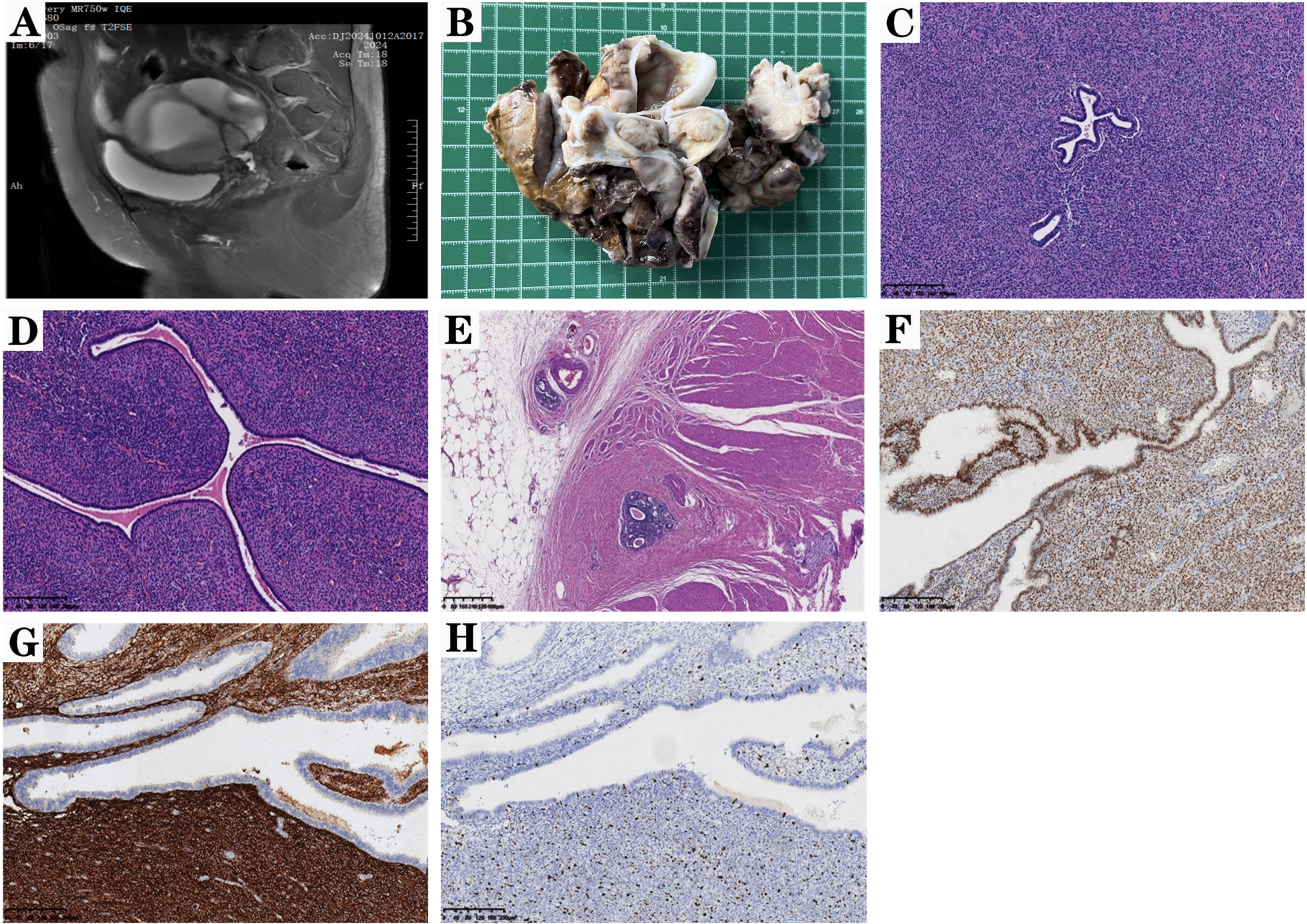
Ovarian Adenosarcoma. **(A)** MRI revealed a complex mass in the left adnexal region. Note: All patient identifiers have been removed from this image to ensure de-identification in compliance with ethical standards and HIPAA regulations. **(B)** Gross examination of the left ovarian cystic-solid mass showed cystic cavities with focal solid components on cross-section. The cut surfaces exhibited variegated coloration (gray-white interspersed with yellow areas) and visible hemorrhagic foci. **(C)** Microscopy demonstrated proliferative glands and stroma. The glandular epithelium displayed simple columnar morphology with cytological blandness, resembling hyperplastic endometrium, while the stromal cells exhibited tightly packed spindle-shaped morphology with mild nuclear atypia (scale bar, 40μm). **(D)** The glandular epithelium was compartmentalized by hypercellular stroma into lobulated configurations, histologically analogous to mammary phyllodes tumor. Characteristic periglandular stromal cuffing was observed (scale bar, 80μm). **(E)** Metastatic deposits in the intestinal wall with associated intravascular tumor emboli (scale bar, 40μm). **(F)** Nuclear ER positivity in both glandular epithelial cells and stromal cells (scale bar, 40μm). **(G)** Cytoplasmic CD10 positivity in stromal cells (scale bar, 40μm). **(H)** Ki67 proliferation index of 20% (scale bar, 40μm).

A 55-year-old female patient was admitted on October 10, 2024. Five days before admission, she had presented to a local hospital with symptoms of nausea and vomiting. During this evaluation, bilateral adnexal mixed masses were discovered incidentally. The patient had no relevant past medical history. Magnetic resonance imaging (MRI) revealed a thickened endometrium and bilateral adnexal complex masses (**Figure 1A**), while contrast-enhanced CT demonstrated right hydroureter and multiple bilateral inguinal lymph node enlargements.

The patient underwent comprehensive staging surgery, including total hysterectomy, bilateral salpingo-oophorectomy, omentectomy, appendectomy, partial rectal resection, partial ureterectomy, and resection of pelvic and abdominal lesions. During surgical exploration, a gray-white cystic-solid mass with a diameter of 10 cm was found within the left ovary, while a 5-cm cystic-solid mass with multiple confluent cysts and dense pelvic wall adhesions was detected in the right ovary. Additionally, a 6×3-cm firm mass encased the pelvic segment of the right ureter, causing dilation of the abdominal ureter (with a diameter of 2 cm), with no abnormalities noted in the left ureter. Two pedunculated dark-red solid masses (with a diameter of 8 cm and 6 cm, respectively) were identified in the presacral rectal mesentery. The appendiceal tip was indurated and densely adherent to colonic mesenteric lesions. Further findings included nodules measuring approximately 1.5 cm in diameter at the right paracolic gutter (umbilical level), an intraumbilical peritoneal mass with a diameter of 3 cm, and omental caking with scattered gray-white nodules ranging from 0.5 cm to 1.5 cm in diameter. No macroscopic lesions were detected on the diaphragmatic, hepatic, or splenic surfaces, although focal peritoneal hyperemia was present. Following surgery, the specimen was fixed in 10% formalin and sent for pathological examination.

### 2.2 Pathological characteristics

Pathological examination revealed bilateral ovarian cystic-solid masses measuring 12 cm ×9.5 cm × 2.5 cm (left) and 3 cm × 2.1 cm × 2 cm (right). The cystic cavities and partially solid cut surfaces exhibited gray-white to yellowish discoloration with hemorrhagic foci (**Figure 1B**). Then, formalin-fixed paraffin-embedded tissue blocks were cut into 4-μm-thick sections and stained with hematoxylin and eosin using a standard protocol.

Microscopically, the tumor was composed of proliferative glands and stroma, with glandular structures separated by abundant stromal components into lobulated architectures resembling breast phyllodes tumors. The glandular epithelium consisted of single-layered columnar cells with bland cytomorphology, mimicking hyperplastic endometrium, while the stromal component featured densely packed spindle cells arranged in fascicles (**Figure 1C**). These stromal cells formed characteristic periglandular cuff-like condensations (**Figure 1D**) around spiral artery-like vessels, exhibiting mild cytological atypia with identifiable mitotic figures. Metastatic tumor involvement was observed in subumbilical lesions, intestinal implants, omentum, and distal right ureter tissue, along with tumor emboli within subserosal vascular channels of the rectum (**Figure 1E**), Confirmed the presence of lymphovascular invasion (LVI).

### 2.3 Immunohistochemistry

Then, immunohistochemical analysis was performed on 4-μm FFPE tissue sections using the following antibodies and conditions: CD10 (Dako, clone 56C6, Ready-to-Use), P53 (Dako, clone DO-7, Ready-to-Use), PCK (Dako, clone AE1/AE3,

Ready-to-Use), WT1 (Dako, clone 6F-H2, Ready-to-Use), Ki67 (Dako, clone SP6, Ready-to-Use), estrogen receptor (ER) (Dako, clone 1D5, Ready-to-Use), progesterone receptor (PR) (Dako, clone PgR 636, Ready-to-Use). All antibodies were detected using the Envision Plus detection system (Dako). Antigen retrieval and staining procedures were performed according to the reagent manufacturer’s instructions. Appropriate positive and negative controls were incorporated throughout the process. Phosphate buffer substituted for the primary antibody served as the negative control, while known positive sections were used as positive controls.

Immunohistochemical analysis demonstrated that glandular epithelial cells were positive for PCK, ER (**Figure 1F**), and PR, while stromal cells exhibited cytoplasmic positivity for CD10 (**Figure 1G**), along with nuclear expression of ER, PR, and WT1. The Ki-67 proliferation index was approximately 20% (**Figure 1H**), and weak P53 positivity was noted, consistent with a wild-type pattern.

Besides, we also evaluated the PD-L1 protein expression (Dako, 22C3, Ready-to-Use) using the Ventana IHC platform;. PD-L1 immunostaining yielded a Combined Positive Score (CPS) of 5.0. Given the absence of established CPS thresholds for ovarian tumors, we referenced the PD-L1 CPS criteria from cervical cancer guidelines. According to relevant clinical guidelines (11), a CPS ≥1 is considered indicative of PD-L1 positivity, suggesting potential responsiveness to PD-L1 inhibitor therapy in such patients.

### 2.4 Next-generation sequencing analysis

To elucidate the molecular profile of this rare tumor, comprehensive genomic profiling was performed. Next-generation sequencing was conducted using a targeted panel approach covering 1150 cancer-related genes (Shengting Group). Genetic testing was performed using high-sensitivity liquid-phase capture coupled with high-throughput sequencing via the MGISEQ-2000 platform.

The sequencing data underwent rigorous quality control and bioinformatic analysis. The analysis achieved an average sequencing depth of 6,460x, an on-target rate of 77.83%, and a Q30 score of 94.98% indicating high-quality data suitable for accurate variant calling. Somatic variants were identified by comparing the tumor sequence data against the GRCh37/hg19 reference genome. Variants in Cancer The interpretation of genetic variants was conducted in accordance with the 2017 standards and guidelines established by AMP, ASCO, and CAP for the interpretation and reporting of sequence variants in cancer (12). To ensure analytical accuracy, the key mutation identified was further validated using high-sensitivity PCR.

Analysis revealed a somatic missense mutation in exon 2 of the GNAQ gene (NM_002072.5): c.286A>T (p.Thr96Ser), with a mutant allele frequency of 6.1%.

### 2.5 Pathological diagnosis

Taken together, these pathological findings confirmed bilateral ovarian adenosarcoma with a low-grade endometrial stromal sarcoma component, notable for lymphovascular invasion (LVI) without perineural involvement. The uterine specimen revealed coexisting endometriosis, while tumor emboli were identified within subserosal vascular channels of the rectum. Metastatic involvement was confirmed in the subumbilical lesions, intestinal implants, omentum, and distal segment of the right ureter.

### 2.6 Treatment and follow-up

The patient initiated oral letrozole therapy on November 8, 2024. Radiotherapy was administered from November 18 to December 26, 2024, with doses of 59.4 Gy/27F to the gross tumor volume (GTV, femoral cavity mass) and 48.6 Gy/27F to the clinical target volume (CTV, pelvic lymph node drainage area). Five cycles of sintilimab immunotherapy were administered from November 27, 2024, to February 28, 2025. During 6 months of follow-up, no evidence of recurrence was detected. Chest CT revealed multiple small pulmonary nodules. However, given the absence of interval growth or significant changes on the recent follow-up scans, and in the context of the patient’s overall stable clinical condition, these nodules were not considered to represent metastatic lesions from the primary ovarian adenosarcoma at this time. The patient was advised to undergo continued active surveillance with CT scans every six months to monitor for any potential changes, in accordance with standard radiological follow-up protocols for indeterminate nodules.

## 3 Discussion

MA is a rare gynecological malignancy characterized by a distinct histopathological admixture of benign glandular epithelium and low-grade sarcomatous stroma (1). Primary ovarian adenosarcoma is extremely uncommon, necessitating rigorous exclusion of endometrial primary tumors as the source of metastasis. In this case, multiple endometrial samplings confirmed the absence of neoplastic components. A multicenter study by Eichhorn et al. involving 40 patients with ovarian MA demonstrated a median onset age of 53 years, with 97.5% (39/40) of cases exhibiting unilateral involvement and only one case (2.5%) presenting with bilateral lesions (stage Ic; left, 9 cm; right, 8 cm) (7). Regrettably, the study lacked detailed documentation of the distinct clinicopathological profile of this bilateral case. Furthermore, this report classified the tumor using the FIGO staging system, with 40 cases being stage I-III (26 stage I, 11 stage II, 3 stage III). It is notable that Stage III cases were exceptionally rare, with only two cases categorized as stage IIIB. In contrast, the clinicopathological features of our case - bilateral primary ovarian involvement and stage IIIC- represent an exceedingly rare presentation, surpassing the rarity of previously reported stage III cases.

Current research suggested that age <53 years, high-grade histology, and high-grade SO (but not low-grade SO) appeared to be associated with recurrence or extraovarian spread (7). To date, no documented cases of ovarian adenosarcoma with LVI have been reported, although a small subset of cases has exhibited postoperative hematogenous metastasis. Daskalaki et al. documented a case of MA with extraperitoneal metastases to the lung, oral cavity, and brain (13). While Carroll et al. noted that uterine adenosarcoma with SO exhibited malignant potential analogous to high-grade sarcoma. Their multivariate analysis identified SO and LVI as predictors of poorer progression-free survival (PFS) and overall survival (OS) (14). A recent study further confirmed LVI as an independent risk factor for disease progression in uterine adenosarcom (14, 15). Notably, our case presented with bilateral primary ovarian adenosarcoma accompanied by extensive metastasis without SO, and LVI was identified here for the first time in this tumor type. These findings propose a novel perspective: LVI could be associated with extraovarian spread in ovarian adenosarcoma. We further hypothesize that LVI may serve as a poor prognostic factor in this rare malignancy. It might explain the poor prognosis observed in a subset of patients without SO.

The pathogenesis of MA remains incompletely understood, with current hypotheses suggesting potential associations with endometriosis, though molecular mechanisms underlying this association are not yet fully elucidated (16). Limited data on MA tumorigenesis indicated that alterations in the *PIK3CA/AKT/PTEN* pathway occurred in 72% of patients (13/18), while the most frequent amplifications involved *MDM2* and *CDK4* (5/18; 28%), suggesting potential therapeutic targets (8). Additionally, *TP53* mutations, although infrequent in MA, have been shown to predict SO development and correlate with aggressive clinical behavior (10) A study has demonstrated that high-grade adenosarcomas exhibited molecular heterogeneity, characterized by genomic instability and *TP53* mutations; and *BAP1* inactivation appeared to be a specific pathogenic driver in a subset of adenosarcomas (9). Notably, we reported the identification of a somatic GNAQ missense variant (NM_002072.5: exon2 c.286A>T, p.Thr96Ser) by NGS in ovarian adenosarcoma. Gαq is a member of the q class of Gα subunits that mediates signals between GPCRs and downstream effectors (17). The GNAQ T96S mutation has been reported in various diseases. Li et al. identified somatic mutations of GNAQ (encoding the T96S alteration of Gαq protein) in 8.7% (11/127) of Natural killer/T cell lymphoma (NKTCL) patients. They found that Gαq suppressed tumor growth of NKTCL via inhibition of the AKT and MAPK signaling pathways (18). Another study reported that transfection with the GNAQ T96S expression vector enhanced anchorage-independent growth, migration, and the MAPK pathways in the SK-Hep-1 cells (19). From a therapeutic perspective, the identification of GNAQ mutation may have clinical implications. A study indicates that GNAQ/11 mutations are prevalent in uveal melanoma, where they drive tumor growth by activating the PKC signaling pathway. As a potent PKC inhibitor, darovasertib is currently under clinical investigation for the treatment of uveal melanoma (20). Therefore, our finding may uncover novel pathogenic mechanisms and provide the rationale for targeted therapy in ovarian adenosarcoma.

Currently, no evidence-based standardized protocol has been established for postoperative adjuvant therapy in MA, and the roles of chemotherapy, radiotherapy, and endocrine therapy remain undefined. For low-grade adenosarcomas without SO but with high ER/PR expression, endocrine therapy represents a potential treatment option (21). Studies have shown that some patients had benefited from leuprolide or anastrozole, suggesting that hormonal-targeted therapy may stabilize disease progression and improve survival (22). In this case, the patient’s ER-positive status and PD-L1 protein expression (CPS = 5.0) informed a treatment regimen of oral letrozole, radiotherapy, and five cycles of sintilimab immunotherapy. Although direct evidence for the application of PD-L1 inhibitors in ovarian adenosarcoma is limited, these inhibitors have demonstrated potential in other gynecological malignancies with similar molecular characteristics. For instance, studies have reported clinical activity of PD-L1 inhibitors in patients with cervical cancer (11). Considering that this particular patient tested PD-L1 positive (Combined Positive Score = 5.0), and presented with a high tumor burden and extensive metastases, an immunocombination therapy regimen was adopted following a multidisciplinary discussion. During the current one-year follow-up period, no recurrence was detected. Chest computed tomography revealed several small pulmonary nodules of uncertain significance; follow-up scans showed no significant changes, and these nodules are currently not considered metastatic lesions from the primary disease. The patient continues to undergo active surveillance.

## 4 Conclusion

We report an exceptionally rare case of ovarian adenosarcoma characterized by bilateral ovarian involvement, staging as IIIC. This case provides well-documented histopathological evidence of LVI, a novel finding in ovarian adenosarcoma. This characteristic finding highlights the need for clinicians to consider risk of postoperative hematogenous metastasis. Moreover, we report the first identification of a somatic *GNAQ* missense variant in this tumor type, though the specific mechanistic role of the *GNAQ* mutation in ovarian adenosarcoma warrants further investigation.

## Data Availability

The original contributions presented in the study are included in the article/supplementary material. Further inquiries can be directed to the corresponding authors.
